# Pollen trapping and sugar syrup feeding of honey bee (Hymenoptera: Apidae) enhance pollen collection of less preferred flowers

**DOI:** 10.1371/journal.pone.0203648

**Published:** 2018-09-12

**Authors:** Tolera Kumsa Gemeda, Jilian Li, Shudong Luo, Huipeng Yang, Tingting Jin, Jiaxing Huang, Jie Wu

**Affiliations:** Key Laboratory for Insect-Pollinator Biology of the Ministry of Agriculture, Institute of Apicultural Research, Chinese Academy of Agricultural Sciences, Beijing, China; Universidade de Sao Paulo Faculdade de Filosofia Ciencias e Letras de Ribeirao Preto, BRAZIL

## Abstract

Pear (*Pyrus bretschneideri*) is characterized by being self-incompatible and dependent on cross-pollination to set fruit. Honeybee (*Apis mellifera*) is considered the most important pollinator of pear. Nevertheless, limited pollen transfer has been cited as the main cause of poor fruit set in many pear orchards. Here, we tested the following hypotheses: (i) colony manipulations increase the pollen collection tendency of honeybees and (ii) the proportion of pollen loads being returned to the hive is from the target plant. The technique reliably and rapidly estimates the pollination of honeybees tested under different colony manipulations: (1) using pollen trapping (PT); (2) PT with sugar syrup feeding (SS) (PTSS); (3) SS alone and (4) control without PT and SS. The results clearly show that the pollen collection of honeybees during the experiment was significantly affected (P < 0.05) by colony manipulations. The mean amount of pollen harvested daily was higher for PTSS (19.4 g) and PT (16.4 g) than for SS (12.85 g) and control (8.7 g) colonies. Therefore, PTSS was the most effective treatment for increasing pear pollen collection; other treatments such as PT and SS could also be useful. This study was important for determining how the behavior of honeybee colonies is shaped through colony manipulation to enhance pollen collection of less preferred pear flowers, which is critical when pollination is required.

## Introduction

Honeybees are well adapted to collect and transfer pollen and thus are the most commonly used crop pollinators. Unlike other flower-visiting solitary insects, honeybees collect large quantities of pollen and nectar [1–5]. Pollen foraging is a collective behavior that is precisely organized and carefully regulated depends on the pollen storage level in the hive, the available resources and the climatic conditions of the area [6–8]. Few studies have examined whether honeybee foraging decisions are sensitive to changes in colony conditions [9–11]. Change in a colony’s pollen reserve can trigger a change in the foragers’ behavior [12,13]. A colony will collect more pollen by recruiting more pollen foragers and increasing the frequency of foraging trips; thus, pollen collection and the pollination of target crops are enhanced [11,14].

*Pyrus bretschneideri*, is widely cultivated in the China due to its great size, good flavor and rich juice [15]. Its flowers offer abundant pollen but produce nectar with a low sugar content [16–19]. This species is self-incompatible and depends highly on cross-pollination to set fruit [20,21]. *Apis mellifera* workers visit pear flowers mainly for pollen for a short duration and often switch to other more attractive plants for nectar when pollination is required [17,20–23]. As a result, pear plant growers are concerned about inadequate fruit yield and forced to hand pollinate each flower, which is labor intensive (personal obs.).

Different colony management techniques were tested to improve honeybee pollination of less attractive crops [24–27]. These techniques require knowledge about the behavior of honeybees and the factors that influence the colony to collect more pollen that enhances pollination [8]. When pollens were removed from colonies, there was an associated increase in the number of pollen foragers, pollen amount and pollen load size [28–30]. Pollen trapping (PT), which removes pollen loads from returning foragers, has been reported to stimulate honeybees to collect more pollen; thereby, target crops could be efficiently visited and pollinated by honeybees [29,31].

Feeding honeybee colonies sugar syrup (SS) within hives has also been shown to increase the total amount of pollen collected [24,32–34]. Sugar syrup feeding enhances pollen collection of colonies, mostly due to changes in the behavior of individual foragers [35]. Sugar syrup has a greater effect on the collection of target crop pollen than on the collection of pollen from other nearby flowers [34].

This work is important for testing the use of colony management that enhances pear pollen collection by honeybees as best measures for pear pollination. The objectives were to determine whether PT and SS colonies would enhance their total pollen collection and pear pollen proportion. This study is expected to provide information that will support pollination researchers and pear growers for successful pear pollination management to improve the fruit set.

## Materials and methods

### Ethics statement

Pear growers that participated in this study were informed about the experiment and provided consent before we started the experiment. Pollen was collected from honeybees with permission from the beekeepers and the land owners.

### Study location and species

The study was conducted in a potential pear-growing area of Yuncheng (N 35.2915, E 110.8461) located in Shanxi Province, China. The area is characterized by monoculture commercial pear plantations where no natural habitats exist for wild pollinators to survive. The orchard has 6.6 ha, and pears were planted with a distance of 6 m between rows and 5 m between plants. The cultivar ‘Suli’ is dominantly planted (93%) in the orchard for commercial fruit production; other cultivars, such as Bali, Yali and Hongxiangsu pear varieties, are usually present as pollinizers and all cultivars flowers at the same time.

The flower morphology of pear consists of a corolla with five petals, each supported by five sepals, that extends to form a spur where nectar is produced. The flower consists of five longer styles associated with a pair of ovules and 21–23 stamens situated below the styles. This morphology indicates that pollinator-mediated cross-pollination is required for successful fruit set.

The blooming time of pear starts in late March and the flowering period ranges from 7 to 10 days but varies with the annual weather conditions. Our field study was conducted from 29 March to 7 April 2016 and from 8 to 17 April 2017 during the peak pear-flowering periods at the study site. Meteorological data such as daily temperature and moisture were recorded. Dandelion (*Taraxacum officinale*), a weed grown in the field and field margins, and peach (*Prunus mongolica*) planted in small patches and/or along borders used as a field demarcation line were grown in the area as competitive flowers that affect the pollination of pear.

### Experimental setup

We brought thirty colonies of *A*. *mellifera* to the pear orchard, and twenty colonies were selected for the experiment and placed in the center of the pear orchard. All colonies were in single Langstroth hives and had equal honeybee populations (approximately 8000–8500 workers), each with two brood frames and two frames with very little pollen and nectar.

Different colony manipulations were tested to determine the response of colonies’ pollen collection behavior. In the first manipulation, five colonies were treated with PT alone; in the second manipulation, five colonies were treated with PT with SS (PTSS); in the third manipulation, five colonies were treated with SS alone; and the control colonies were not treated with PT or SS.

To minimize the environmental factors, we assigned colonies randomly among treatments and conducting experiments with brief periods of time in the same location. We collected pollen using PT in which honeybees entered the hive through pollen traps whose mesh grid dislodged 70% of the pollen loads from the hind-leg [36]. Pollen fell and was stored in a tray covered by a screen that allowed pollen but not the bees to pass. Before pollen collections, SS was carried out each evening by placing 200 ml (50% water and 50% white sugar) in the feeder, which was placed in the hives.

### Colony pollen collection

The total numbers of pollen foragers were examined to determine if hive manipulations affects in colony level response. Honeybees’ entering the hive with pollen loads were counted from 8:00 to 17:00 within 5 minutes at one-hour intervals using a digital video camera. We also measured the wet weight of pollen collected four times a day from 9:00 to 17: 00 at two-hour intervals and compared these values among the treatments. The pollen weight under the non-PT treatments (SS and control) was estimated from the number of honeybees entering with pollen (two pollen pellets per bee), considering the mass of each pollen load and the 70% trapping efficiency of PT within a given interval.

### Individual pollen collection

The mass of pollen loads (pellets) was collected to investigate the individual pollen foraging behavior of honeybees. In each interval, bees with pollen were captured, pollens were carefully dislodged from the hind legs, and the weight was recorded and compared across days and times.

### Pollen proportion

We randomly collected samples of pollen on each experimental day; five grams of pollen samples were taken and sorted by color. Visual identification of pollen color and comparison with reference specimens is the standard approach in pollination ecology [37]. Pollen of each color was fixed on separate slides and identified under 400x magnification following previously described methods [36]. Pollen of each color was identified to the species level by comparing the morphologies of pollen grain reference collected from the anthers of surrounding plant species in bloom. We calculated the proportion of pollen by weight identified as belonging to the target species. Pollen from all pear varieties was identified as *Pyrus* sp.

## Data analysis

Statistical analysis was performed using the R-Project software (version 3.3.2). Data were analyzed using ANOVA to investigate the effects. All data were tested for the assumption required by ANOVA, and when the data did not meet the assumptions, the appropriate log transformation was performed. For all data tables, mean values are listed plus or minus with the standard error. Response variables were pooled if necessary, and the mean number of bees foraging pear flowers, mean number of bees entering with pollen, pollen weight and pollen proportion were analyzed. Post hoc analysis was carried out using Tukey’s test for comparison of means at P < 0.05 significance level. Sigma-Plot (version 12.5) was used to sketch graphs.

## Results

### Pollen collection of honeybees

Pollen collection was estimated by quantifying the number of honeybees entering with pollen. The number of honeybees entering with pollen was similar between study years, with no significant variations (F_1, 238_ = 1.78, P = 0.18). However, pollen collection varied among experimental treatments (F_3, 716_ = 53.8, P < 0.001), days (F_2, 717_ = 21.4, P < 0.001) and times of day (F_9, 710_ = 25.1, P < 0.001).

**Fig 1:**
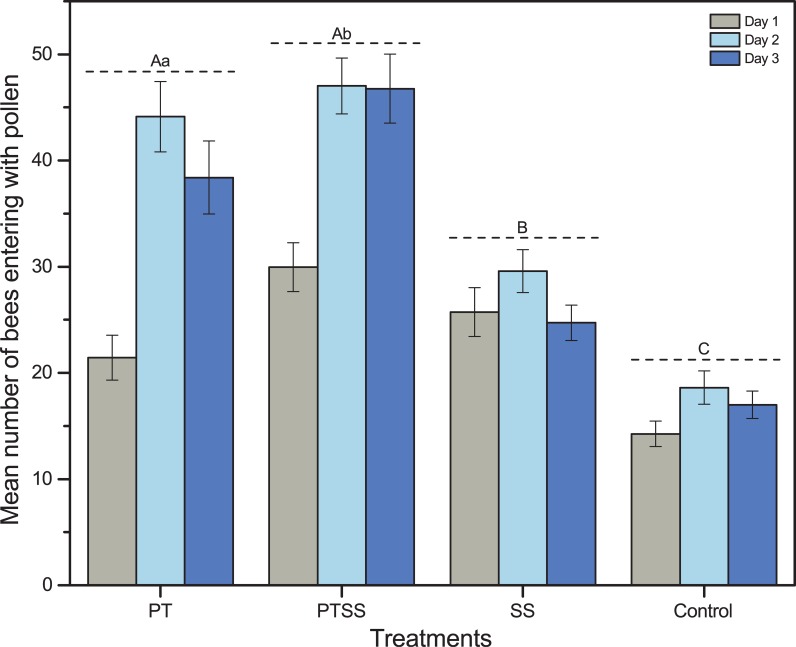
Mean number of honeybees entering with pollen. Bars show the mean number of honeybees (mean ± se) entering with pollen under different treatments (i.e. mean number of pollen foragers returning within 5 min). Plotted data are means and standard errors indicate significant differences. Post hoc testing created the groups that capital letters indicate the significance level P < 0.01 and small letters indicate the significance level P < 0.05.

**Fig 2:**
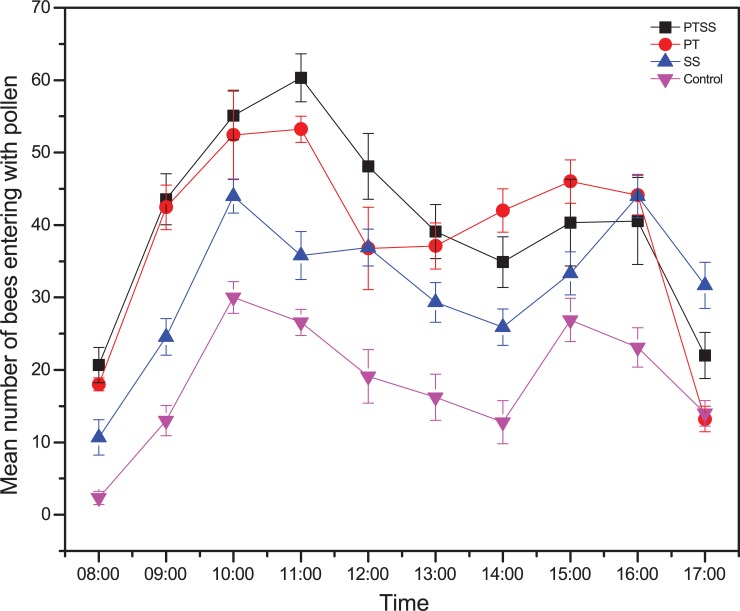
Mean number of bees entering with pollen over time. Figures show the pollen-foraging rate measured as the number of foragers returning to the hive (mean ± se) within a 5-min period over time. The figures show similar pattern with different intensities because of the treatment effect.

The mean number of pollen foragers returning to the hive per 5 min was higher for PTSS (41 ± 1.6 bees) and PT (35 ± 1.8 bees) than for SS (27 ± 1.67 bees) and control (17 ± 0.79 bees) colonies. Pollen collection increased with days and was influenced by the times of day. The mean number of pollen foragers was interestingly higher on the second day of pollen collection for all treatments (Fig 1). Pollen collection peaked at 11:00, decreased from 11:00 to 14:00 and experienced a minor peak from 15:00 to 16:00 (S1 Table, Fig 2).

### Pollen weight

Pollen weight was inferred from the pollen loads collected through pollen traps. The pollen weight was not significantly varied between years (F_1, 238_ = 2.31, P = 0.13), and the average values were 17 ± 1.2 g and 19.1 ± 1.0 g in year 1 and year 2, respectively. ANOVA from year 1 and year 2 pooled together showed that pollen weight was significantly different among experimental treatments (F_3, 238_ = 3.8, P = 0.02), days (F _2, 237_ = 20.06, P = 0.002) and times of day (F_3, 236_ = 8.25, P < 0.001). However, Tukey’s means comparison test showed that there were no significant differences in pollen weight (F_1, 238_ = 3.49, P = 0.63) between PTSS and PT treatments. Colonies under PT, PTSS or SS treatment collected relatively more pollen, with mean weights of 16.4 ± 1.1 g, 19.4 ± 1.2 g and 12.85 ± 1.1 g, respectively, compared to control group (8.7 ± 0.83 g).

**Fig 3:**
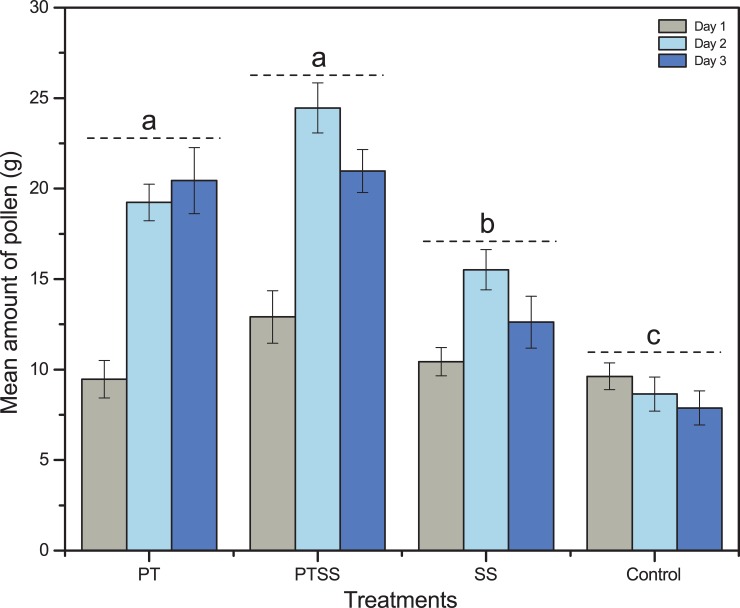
Mean amount of pollen weight in different treatments. Bars show pollen weight was significantly different among treatments (mean ± se). Post hoc testing created the groups, and letters indicate statistically significant differences at P = 0.05 level.

**Fig 4:**
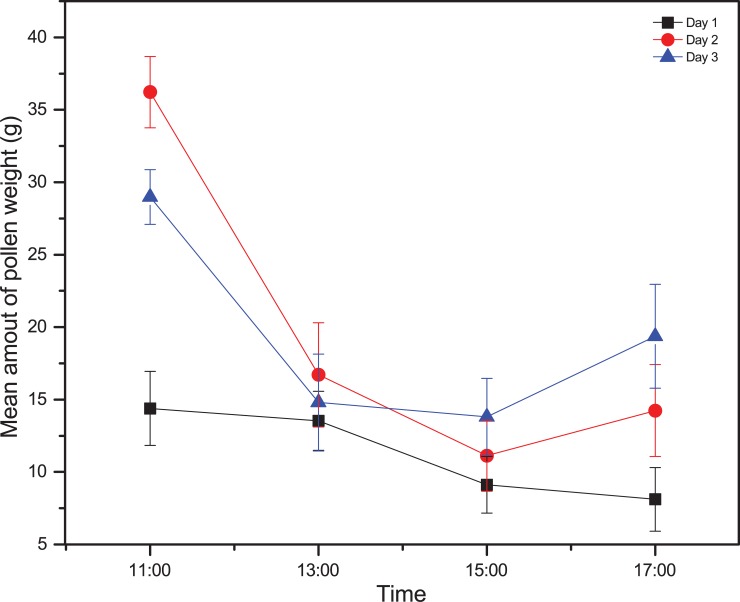
Mean amount of pollen weight measured at different time of days. Figures are pollen weight across time of different days (Day 1, Day 2 and Day 3). Plotted data are means and standard errors significant differences at P = 0.05 level.

Pollen weight was low on day 1 (9.47 ± 1.0 g, 12.9 ± 1.4 g, 10.4 ± 0.78 g and 9.62 ± 0.74 g for PT, PTSS, SS and control colonies, respectively). The pollen weight of the PT and PTSS colonies surprisingly increased on day 2 (19.23 ± 1.0 g and 24.45 ± 1.38 g, respectively). On day 3, the pollen weight was relatively stable compared to day 2, with mean pollen weights of 20.44 ± 1.8 g, 20.97 ± 1.2 g, 12.6 ± 1.4 g and 7.88 ± 0.94 g for the PT, PTSS, SS and control colonies, respectively (Fig 3). The pollen weight was highest before late afternoon, between 9:00 and 11:00, during which 33% of the total pollen was collected, and slightly decreased with time (S2 Table, Fig 4).

### Pollen pellet weight

**Fig 5:**
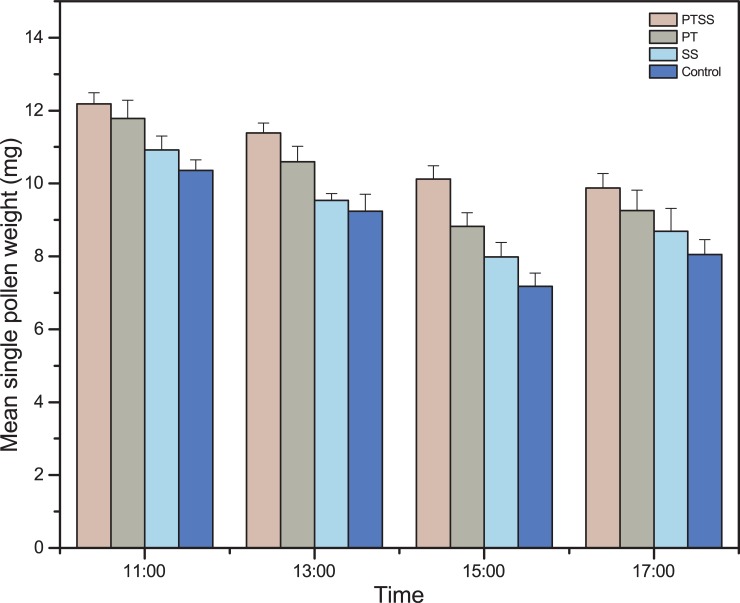
Mean amount of single pollen pellet weights collected from pollen foragers. Bars show that weight of single pollen load (mean ± se.) varied with treatments and times. Single pollen pellets obtained from PTSS treatment were highest at all time points and decreasing with time.

The weight of each pollen pellet was measured, and the mean ranged from 4.20 to 13.68 mg/pellet. The pollen pellet weight was significantly varied among treatments (F _3,140_ = 6.7, P <0.001), and times of day (F _3,140_ = 22.5, P < 0.001), but not significantly varied across days (F _2,141_ = 0.031, P = 0.98). The weight of single pollen load was highest for PTSS (10.62 ± 0.27 mg), followed by PT (10.11 ± 0.29 mg), SS (9.05 ± 0.29 mg) and control (8.85 ± 0.41 mg). It decreased with time of day, with mean values of 11.31 ± 0.26 mg, 10.18 ± 0.27 mg, 8.31 ± 0.32 mg and 8.14 ± 0.21 mg at 11:00, 13:00 15: 00 and 17:00, respectively (S3 Table, Fig 5).

### Pollen proportion

**Fig 6:**
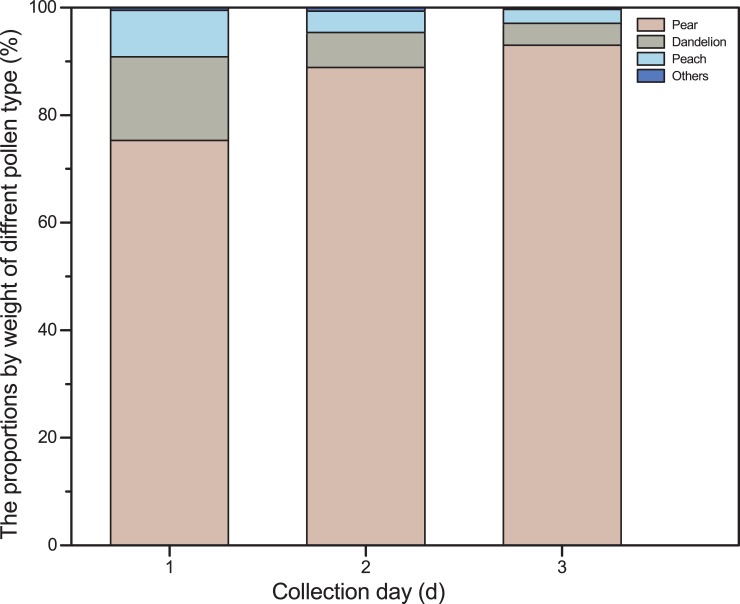
The proportions of different pollen types for day 1, day 2 and day 3. Pollen proportions for pear, peach and dandelion species were plotted on the bars. The bars show that the proportion of pollens from pear increases with days whereas the proportion of pollens from peach and dandelion decreases with days.

Other plant species were present in the study area; dandelion (*T*. *officinale*) and peach (*P*. *mongolica*) were the most common competitive flowering species. The mean proportion of pollen that honeybees collected from the target crop (pear) by weight was highest (85.7%), followed by peach pollen (8.7%), dandelion pollen (5.1%) and others (0.5%). Moreover, the proportion of pear pollen collected by weight significantly increased (F_2, 57_ = 37.0, P < 0.001) across days and was highest on day 3. The proportion of pear pollen collected by weight was 75.3% on the first day and increased to 88.8% on the second day and 93.2% on the third day. On the other hand, the proportion of peach and dandelion pollen collected by weight decreased as the days advanced (S1 Fig, Fig 6).

## Discussion

The behavior of pollen foraging is one of the functional flexibility in social insects, which is important in planning management of honey bee colonies for pollination of target crops [6]. Our study adds to the understanding of pollination management of honey bee colonies for less preferred plant through detail description of pollen collecting behavior of foragers under different colony manipulations.

The results from this study highlight that colony manipulation for pollination that merit further attention. First, *A*. *mellifera* foragers visit pear flowers for pollen in shorter duration and switches to other more attractive plants for nectar. These findings support other research that being brought to orchards to pollinate specific crops, *A*. *mellifera* did not always collect pollen from those crops unlike native bee pollinators [36]. Second, the behavior of honeybee colonies is shaped through colony manipulations to enhance pollen collection of less preferred pear flower when pollination is required. Third, the proportion of pear pollen collected by forager bees were increased as PTSS was continued possibly enhanced the less preferred pear pollen collection.

Our results clearly demonstrate that colony manipulations using PT and PTSS increased the daily mean number of pollen foragers and pollen weight of the colonies. The most important finding was that the proportion of pear pollen of the total pollen collected increases. Colonies regulate their pollen-foraging activities based on the amounts of stored pollen and the number of young broods in the hive [29]. Our data support earlier works that colonies with a small pollen reserve are suddenly supplied with a large pollen reserve at this time, the number of bees engaged in pollen collection starts to fall. Conversely, when colonies are forced to deplete their pollen reserves, the number of forager bees engaged in pollen collection rises [6,11,38,39]. This argument was supported by our results that PT reduced the pollen store in the colony and significantly induced the honeybees to collect more pollen from available plant resources (pear). This created a greater chance of the bees coming into contact with pear flowers, thereby increasing the chance of pear pollen collection and its pollination.

Studies on other plant species confirmed that removing pollen from a colony can trigger a change in the foraging behavior of honeybees, resulting in increased the target plant pollen collection and pollination [29,31,40,41]. In a kiwifruit orchard, PT increases the daily pollen collection of colony by 16.6%, which is an important practice to enhance kiwifruit pollination [41].

Pollen collectors are top foragers that have better stigma contact when pollination is required than do nectar collectors that collect nectar from the flower base (side workers) [11,33,42]. If colonies are manipulated to deplete their pollen reserves and stimulated by SS feeding, they immediately responded by increasing the number of pollen foragers [33,35,38]. During this time, honeybees immediately increase their pollen gathering from all available plants, rather than choosing pollen with higher protein content [40]. The assumption that pollen depletion by PT enhances the mobility of honeybees in pear orchard to fulfill the deficiency in the colony is important in our study. This mechanism enhances pear pollen collection frequency of honeybees from the top of flower, which ensures the stigma contact by honeybees and enhances the possibility of cross pollination.

Sucrose is an important factor used as a primary signal reward to induce honeybees to collect more pollen [43]. The low visitation of *A*. *mellifera* to pear flowers might be due to the low sucrose content of the flowers’ nectar [16]. Sugar syrup feeding is likely to either divert honeybees that were visiting other flowers to the target plant or discourage them from collecting more nectar and encourage them instead to collect more pollen [24,32,35].

Sugar syrup feeding increased sweet cherry pollen collection by 2.16-fold, field bean pollen collection by 3.27-fold, red clover pollen collection by 5.2-fold and kiwifruit pollen collection by 7.9-fold [32,35]. In our results, PTSS increased the number of pollen foragers, which might result in more honeybees foraging on pear flowers, thereby improve the possibility of pear pollen collection and pollination.

Pollen collection behavior of individual worker bees is crucial for determining the collective behavior of colonies [6]. Pollen load size collected by individual honeybee are affected by a number of factors such as pollen amount in the colonies, time of day, and amount pollen produced by plant species, and meteorological conditions of the area [41]. Our results clearly demonstrate that pollen load (pellet) weight varies with colonies manipulations and pollen collection time. It is clear that honeybees with larger pollen load visit more flowers to get their full load and possibly enhance pear pollination [27]. However, it is not a reliable measure of pollination efficiency of honeybee and need further investigation.

Certain plants in the vicinity of a pear orchard may be more attractive to honey bees than pear flower, because they produce more nectar or pollen[44]. As a result, honey bees will neglect the target crop. The problem of plant competition for bee visits has been reported for several crops [10,26,44–46]. This study determines Dandelion (*T*. *officinale*) and peach (*P*. *mongolica*) are found as competitive species grown in pear orchard. Dandelion was sparsely grown in the pear orchard, and the proportion of pollen collected during PT decreased after midday on warm days. On the other hand, honeybees frequently visited peach flowers throughout the day, but the peach plant density in the orchard was too low to significantly compete with pear flowers. Accordingly, the effect of competitive species may be less important as PTSS is continued. Removal of other competitive flowering plant species from pear orchard should significantly enhance the collection of pear pollen and the pollination efficiency of honeybees.

In conclusion, pear flowers have short flowering duration, which requires designed pollination to efficiently utilize honey bees. Honey bee colonies adaptively alter pollen collection behavior responding to colony managements. Given the described behavior of honey bees, that do not prefer for pear flower, the use of pollen traps with sugar syrup feeding increases pear pollen collection and probably its pollination. The techniques can serve as rapid and inexpensive pollination management of honey bee colonies and easily applied by pear growers. Beekeepers might be benefited from renting colonies and pear growers are likely to enhance pear pollination services for optimum pear fruit production.

## Supporting information

S1 Table Number of honeybees entering with pollen. (XLSX)

S2 Table Pollen weight (g) during pollen trapping. (XLSX)

S3 Table Single pollen load (pellet) weight in milligram. (XLSX)

S1 Fig Different color of pollen collected by honey bees. (TIF)

## References

[1] Stern R, Sapir G, Shafir S, Dag A, Goldway M (2007) The appropriate management of honey bee colonies for pollination of Rosaceae fruit trees in warm climates. Middle East Russian Journal Plant Science Biotechnology 1: 13–19.

[2] Müller A, Diener S, Schnyder S, Stutz K, Sedivy C, et al (2006) Quantitative pollen requirements of solitary bees: Implications for bee conservation and the evolution of bee-flower relationships. Biological Conservation 130: 604–615.

[3] Wolf S, Lensky Y, Paldi N (1999) Genetic variability in flower attractiveness to honeybees (*Apis mellifera* L.) within the genus *Citrullus*. HortScience 34: 860.

[4] Aleixo KP, Menezes C, Imperatriz Fonseca VL, Da Silva CI (2017) Seasonal availability of floral resources and ambient temperature shape stingless bee foraging behavior (Scaptotrigona aff. depilis). Apidologie 48: 117–127.

[5] Panday D. Honeybees, their pollination behavior and relation to livelihoods. In: Jha PK, Shrestha KK, Chaudhary RP, Shrestha BB, editors; Proceedings of International Conference on Biodiversity, Livelihood and Climatic Change. Kathmandu; 2015. pp. 197–208.

[6] Weidenmller A, Tautz J (2002) In-hive behavior of pollen foragers (*Apis mellifera*) in honey bee colonies under conditions of high and low pollen need. Ethology 108: 205–221.

[7] Pankiw T, Page RE Jr, Kim Fondrk M (1998) Brood pheromone stimulates pollen foraging in honey bees (*Apis mellifera*). Behavioral Ecology and Sociobiology 44: 193–198.

[8] de Lima EG, Cristina Camargo S, Rosa Santos PD, Santos Oliveira JW, Arnaut De Toledo VDA (2016) Regulation of pollen foraging activity in *Apis mellifera* africanized honeybees colonies. Agricultural Sciences 7: 335–340.

[9] Wolf TJ, Schmid-Hempel P (1990) On the integration of individual in honeybees in social insects: nectar-collection ergonomics. Behavioral Ecology & Sociobiology 27: 103–111.

[10] Erickson EH, Whitefoot LO, Kissinger WA (1973) Honey bees: A method of delimiting the complete profile of foraging from colonies. Environmental Entomology 2: 531–536.

[11] Seeley TD (1997) The wisdom of the hive: the social physiology of honey bee colonies Cambridge, Massachusetts London: Harvard University Press. 295 p.

[12] Dogterom M, Markl W (1999) Pollen storage and foraging by honey bees (Hymenoptera: Apidae) in highbush blueberries (Ericaceae), cultivar bluecrop. Canadian Entomologist 131: 757–768.

[13] Dreller C, Tarpy DR (2000) Perception of the pollen need by foragers in a honeybee colony. Animal Behaviour 59: 91–96. 10.1006/anbe.1999.1303 10640370

[14] Cortopassi-Laurino M, Imperatriz-Fonseca VL, Roubik DW, Dollin A, Heard T, et al (2006) Global meliponiculture: Challenges and opportunities. Apidologie 37: 275–292.

[15] Qin G, Tao S, Zhang H, Huang W, Wu J, et al (2014) Evolution of the aroma volatiles of pear fruits supplemented with fatty acid metabolic precursors. Molecules 19: 20183–20196. 10.3390/molecules191220183 25474290 PMC6271835

[16] Gemeda TK, Shao Y, Wu W, Yang H, Huang J, et al (2017) Native honey bees outperform adventive honey bees in increasing *Pyrus bretschneideri* (Rosales: Rosaceae) pollination. Journal of Economic Entomology 110: 2290–2294. 10.1093/jee/tox286 29126172

[17] Benedek P, Ruff J (1998) Flower constancy of honeybees and its importance during pear pollination. Acta Horticulturae: 427–428.

[18] Farkas A, Oroszkovács Z, Szabó LG (2002) Insect attraction of flowers in pear cultivars. Acta Horticulturae 14: 773–776.

[19] Ma WH, Shao YQ, Zhao HT, Tian SH, Meng J, et al (2015) Using bee attractants to improve honeybee foraging on Dangshan pear (*Pyrus communis* L.). Journal of Agricultural Science & Technology 17: 1551–1558.

[20] Maccagnani B, Ladurner E, Santi F (2003) *Osmia cornuta* (Hymenoptera, Megachilidae) as a pollinator of pear (*Pyrus communis*): fruit- and seed-set. Apidologie 34: 207–216.

[21] Quinet M, Warzée M, Vanderplanck M, Michez D, Lognay G, et al (2016) Do floral resources influence pollination rates and subsequent fruit set in pear (*Pyrus communis* L.) and apple (Malus x domestica Borkh) cultivars? European Journal of Agronomy 77: 59–69.

[22] Monzon H, Bosch J, Retana J (2004) Foraging behavior and pollinating effectiveness of *Osmia cornuta* (Hymenoptera: Megachilidae) and Apis mellifera (Hymenoptera: Apidae) on“Comice”pea. Apidologie 35: 575–585.

[23] Webster A (2002) Factors influencing the flowering, fruit set and fruit growth of European pears. Acta Horticulturae 596: 699–709.

[24] Goodwin RM (2015) Feeding sugar syrup to honey bee colonies to improve pollination: a review. Bee World 78: 56–62.

[25] Benedek P (2003) Bee pollination of fruit trees: Recent advances and research paper activity II. Journal of Apicultural Research 47: 95–101.

[26] Malerbo-Souza DT, Nogueira-Couto RH, Couto LA (2004) Honey bee attractants and pollination in sweet orange, Citrus sinensis, Var. Pera-Rio. Journal of Venomous Animals and Toxins including Tropical Diseases 10: 144–153.

[27] Roman A (2004) Pollen hoarding in the late summer season by honeybee (*Apis mellifera* L.) colonies. Journal of Apicultural Science 48: 37–45.

[28] Dreller C, Page RE Jr., Fondrk MK (1999) Regulation of pollen foraging in honeybee colonies: effects of young brood, stored pollen, and empty space. Behavioral Ecology and Sociobiology 45: 227–233.

[29] Webster T, Thorp R, Briggs D (1985) Effects of pollen traps on honey bee (Hymenoptera: Apidae) foraging and brood rearing during Almond and Prune pollination. Environmental Entomology 6: 683–686.

[30] Stern R, Eisikowitch D, Dag A (2015) Sequential introduction of honeybee colonies and doubling their density increases cross-pollination, fruit-set and yield in‘Red Delicious’apple. The Journal of Horticultural Science and Biotechnology 76: 17–23.

[31] Barker RJ (1971) The influence of food inside the hive on pollen collection by a honeybee colony. Journal of Apicultural Research 10: 23–26.

[32] Goodwin RM, Houten AT (1991) Feeding sugar syrup to honey bee (*Apis mellifera*) colonies to increase kiwifruit (*Actinidia deliciosa*) pollen collection: effects of frequency, quantity and time of day. Journal of Apicultural Research 30: 41–49.

[33] Goodwin RM (1986) Increased kiwifruit pollen collection after feeding sugar syrup to honey bees within their hive. New Zealand Journal of Experimental Agriculture 14: 57–61.

[34] Goodwin RM, Ten Houten A, Perry JH (1991) Effect of variations in sugar presentation to honey bees (*Apis mellifera*) on their collection of kiwifruit (*Actinidia deliciosa*) pollen. New Zealand Journal of Crop and Horticultural Science 19: 259–262.

[35] Free J (1965) The behaviour of honeybee foragers when their colonies are fed sugarsyrup. Journal of Apicultural Research 4: 85–88.

[36] Pettis JS, Lichtenberg EM, Andree M, Stitzinger J, Rose R, et al (2013) Crop pollination exposes honey bees to pesticides which alters their susceptibility to the gut pathogen *Nosema ceranae*. PLoS ONE 8: e70182 10.1371/journal.pone.0070182 23894612 PMC3722151

[37] Wilson EE, Sidhu CS, LeVAN KE, Holway DA (2010) Pollen foraging behaviour of solitary Hawaiian bees revealed through molecular pollen analysis. Molecular Ecology 19: 4823–4829. 10.1111/j.1365-294X.2010.04849.x 20958818

[38] Fewell J, Winston M (1992) Colony state and regulation of pollen foraging in the honey bee, *Apis mellifera* L. Behavioral Ecology and Sociobiology 30: 387–393.

[39] Danka RG, Hellmich R, Rinderer T (1987) Diet-selection ecology of tropically and temperately adapted honey bees. Animal Behaviour 35: 1858–1863.

[40] Pernal SF, Currie RW (2001) The influence of pollen quality on foraging behavior in honeybees (Apis mellifera L.). Behavioral Ecology & Sociobiology 51: 53–68.

[41] Goodwin RM, Perry JH (1992) Use of pollen traps to investigate the foraging behaviour of honey bee colonies in kiwifruit orchards. New Zealand Journal of Crop and Horticultural Science 20: 23–26.

[42] Sapir G, Baras Z, Azmon G, Goldway M, Shafir S, et al (2017) Synergistic effects between bumblebees and honey bees in apple orchards increase cross pollination, seed number and fruit size. Scientia Horticulturae 219: 107–117.

[43] Aronne G, Giovanetti M, Guarracino MR (2012) Foraging rules of flower selection applied by colonies of *Apis mellifera*: ranking and associations of floral sources. Functional Ecology 26: 1186–1196.

[44] Olsen LG, Hoopingarner R, Martin EC (2015) Pollen preferences of honeybees sited on four cultivated crops. Journal of Apicultural Research 18: 196–200.

[45] Free JB (1968) Dandelion as a competitor to fruit trees for bee visits. The Journal of Applied Ecology 5: 169.

[46] Benedek P, Beres I, Nyeki J (1998) Competition between pear flowers, flowering weeds and other fruit trees for honeybee pollination. Acta Horticulturae: 417–426.

