# Supplementary figures and images for: Pollen trapping and sugar syrup feeding of honey bee (Hymenoptera: Apidae) enhance pollen collection of less preferred flowers
Source: PLoS One. 2018 Sep 12;13(9):e0203648. doi: 10.1371/journal.pone.0203648 (PMC6135515; doi:10.1371/journal.pone.0203648)

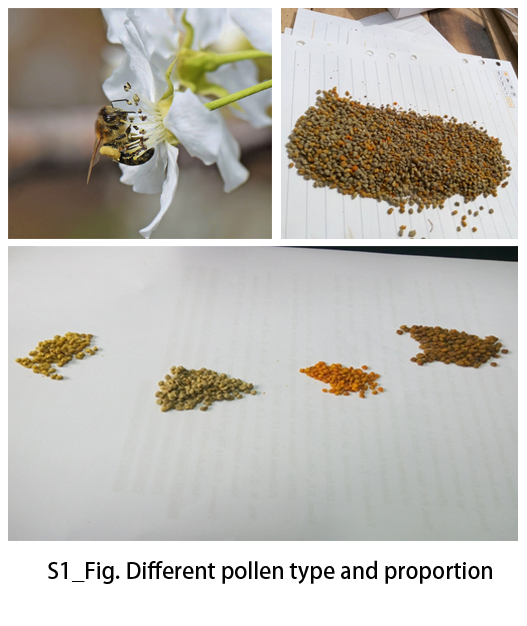

Supplement: S1 Fig — (TIF) [file pone.0203648.s004.tif]
